# Current Trends and Challenges of Fecal Microbiota Transplantation—An Easy Method That Works for All?

**DOI:** 10.3390/biomedicines10112742

**Published:** 2022-10-28

**Authors:** Cátia Almeida, Rita Oliveira, Pilar Baylina, Rúben Fernandes, Fábio G. Teixeira, Pedro Barata

**Affiliations:** 1 LaBMI—Laboratory of Medical & Industrial Biotechnology, Porto Polytechnic Institute, 4200-375 Porto, Portugal; 2 Department of Biomedicine, Unit of Biochemistry, Faculty of Medicine of Porto University, 4200-319 Porto, Portugal; 3 FP-i3ID, HEFP, FCS-UFP—Fernando Pessoa Hospital, Faculty of Health Sciences, Fernando Pessoa University, 4200-150 Porto, Portugal; 4 ESS-IPP—Health School, Porto Polytechnic Institute, 4200-072 Porto, Portugal; 5 i3S—Instituto de Investigação e Inovação em Saúde, Universidade do Porto, 4200-135 Porto, Portugal; 6 ICVS/3B’s-PT Government Associated Lab, 4710-057/4805-107 Braga/Guimarães, Portugal; 7 Life and Health Sciences Research Institute (ICVS), School of Medicine, University of Minho, 4710-057 Braga, Portugal

**Keywords:** fecal microbiota transplantation, gut microbiota, neurological disorders, cardiometabolic disorders, gastrointestinal disorders, cancer

## Abstract

The gut microbiota refers to bacteria lodges in the gastrointestinal tract (GIT) that interact through various complex mechanisms. The disturbance of this ecosystem has been correlated with several diseases, such as neurologic, respiratory, cardiovascular, and metabolic diseases and cancer. Therefore, the modulation of the gut microbiota has emerged as a potential therapeutic tool; of the various forms of gut microbiota modulation, fecal microbiota transplantation (FMT) is the most approached. This recent technique involves introducing fecal material from a healthy donor into the patient’s gastrointestinal tract, aiming to restore the gut microbiota and lead to the resolution of symptoms. This procedure implies a careful donor choice, fine collection and handling of fecal material, and a balanced preparation of the recipient and consequent administration of the prepared content. Although FMT is considered a biological therapy with promising effects, side effects such as diarrhea and abdominal pain have also been claimed, making this a significant challenge in the application of FMT. Bearing this in mind, the present review aims to summarize the recent advances in understanding FMT mechanisms, their impact across different pathological conditions, and the associated side effects, emphasizing the most recent published data.

## 1. Introduction

For almost a century, it has been recognized that human beings have a diverse and dense ecosystem of microorganisms, which is currently called the human microbiome, where the number of bacterial cells exceeds the total number of human cells and has a progressive distal increase in bacteria along the gastrointestinal tract (GIT). However, we are still beginning to understand how these microorganisms behave and play a role in human health [1].

The human microbiota comprises more than 100 trillion different microbial species, within which bacteria, fungi, viruses, archaea, and protozoa can be distinguished [2]. These microorganisms and their genes form a very dynamic microbial community living in different areas of the human body and playing a vital role in the host’s health [1,3]. *Bacteroidetes*, *Firmicutes*, *Actinobacteria*, and *Proteobacteria* are four of the most abundant phyla in the human microbiome, with the first two representing together 90% of the microbiota composition [4,5]. Indeed, from the functional and anatomical point of view, the composition of an adult human microbiome remains practically stable and resilient over time, as its great microbial diversity is acquired during and after birth [6]. Nevertheless, microbiome composition can be changed at any time in an individual’s life by targeted and untargeted interventions [7], such as diet, probiotics, prebiotics, viruses, host genetics, and drugs [6]; bio-engineered commensals and drugs targeting selected microbial metabolism [2]; and bacteriophage therapy and CRISPR-Cas9 [8,9]. Additionally, studies have claimed that gut microbiome composition can also be mainly shaped by environmental factors [10]. Actually, this complex ecosystem has already been recognized for many functions, with active participation in human homeostatic mechanisms, such as nutrient metabolism, maintenance of the integrity of the intestinal mucosa barrier, satiety regulation, defense against pathogens, and development of the immune system [1,11].

In the GIT resides the most significant number and diversity of microorganisms [12], and they coexist in harmony with their host, demonstrating a symbiotic relationship [8]. The gut microbiota affects not only the intestine but also several other areas, such as the heart, liver, kidneys, lungs, and even the immune system [12,13]. Although a balance should be respected between the microbiome and its host to optimize metabolic and immune functions, there is no ideal composition since each individual has a distinct microbiome [14]. Nevertheless, evidence for the importance of a stable microbiome today is unmistakable [1,15]. Many studies support the idea that certain bacterial species in the digestive tract have pathogenic potential, while others are protective and prevent colonization through several recently discovered mechanisms [16,17,18]. Therefore, when dysregulation of the human microbiota occurs, such process is called dysbiosis, which in certain diseases could represent the pathophysiological onset [3]. Indeed, it has been shown that bacteria can appear in different areas of the human body and can communicate with each other bidirectionally, thereby indicating that a vital crossover dialogue between the mucous membranes of the human body (both in a healthy and a pathological state) may exist, since the presence of intestinal complications has been demonstrated in respiratory, cardiac and central nervous system diseases [19]. Therefore, with the present review, we intend to hypothesize fecal microbiota transplantation (FMT) as a prominent method to reshape gut microbiota, describing its positive changes in the progression of diseases, and to explore how the exposure to the donor’s microbiota may change the immunological tone arising from the intestinal wall by addressing some critical issues, such as the following: (1) To what extent does the recipient shift their microbiome to a more favorable phenotype? (2) How does key species engraftment taking place determine the recipient’s success?

**Figure 1:**
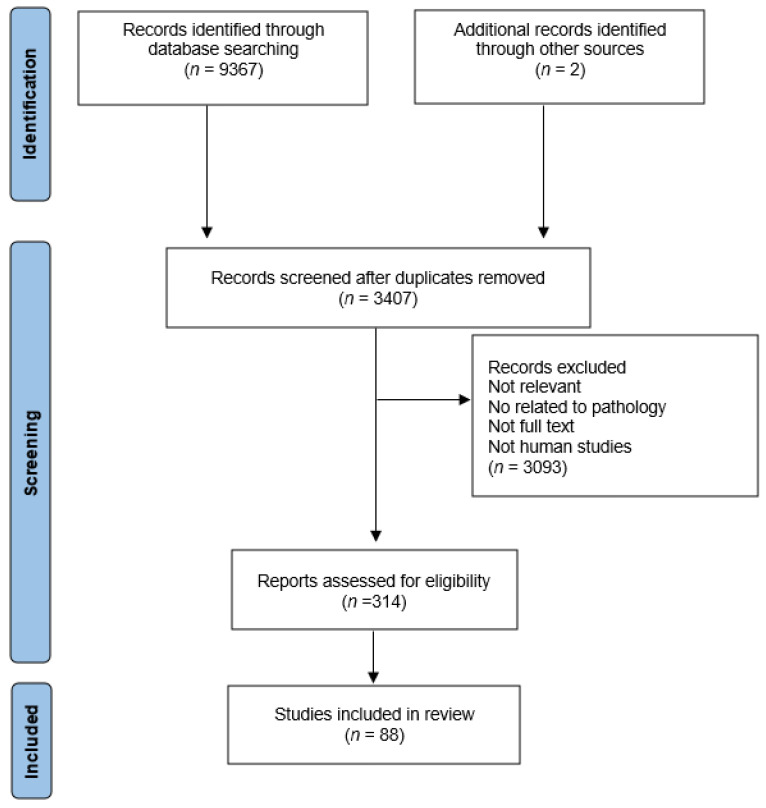
PRISMA flow diagram for the scoping review.

Bearing all this in mind, in addition to the need to clarify the microbiome’s role in physiological and pathological conditions, it also becomes important to explore strategies or therapies that can modulate it, as is the case of FMT procedures. For that, in the scope of this review, we will discuss the current understanding of FMT by reviewing recent experimental data addressing FMT’s potential and efficiency in treating multiple diseases, namely neurological, cardiometabolic, gastrointestinal, and respiratory disorders and cancer [20]. Therefore, using different electronic databases, including PubMed and Web of Science, from conception to July 2022, the purpose was to conduct a literature search to locate relevant papers using the keywords ‘Fecal Microbiota Transplantation’ (and their respective correlation) with ‘Gut Microbiota’, ‘Neurological Disorders’, ‘Cardiometabolic Disorders’, ‘Gastrointestinal Disorders’, and ‘Cancer’ (see Figure 1). Based on this, studies had to satisfy the following requirements to be included in this review: studies related to one of the pathologies mentioned above, full text available, human or animal studies, and a control group receiving a placebo or an autologous FMT. Studies with patients receiving FMT through different modalities, such as colonoscopy, enema, or enteric tubes, were all considered for this review (Figure 1). Furthermore, each one of the retrieved papers was evaluated for relevance and cross-references, and duplicates were eliminated.

## 2. Fecal Microbiota Transplantation

Nowadays, FMT is used in humans and animals as a way of treatment for numerous conditions associated with gut dysbiosis or during scientific experiments to study the role of the microbiota in organisms [3]. Experimentally, this method involves transferring the intestinal bacterial community from a previously studied healthy donor, or from the recipient in a specific period before the onset of the disease and associated dysbiosis, to a recipient who has an imbalance in their gut microbiota [3,20].

Several studies reported that this strategy reduces mortality among severe *Clostridium difficile* infection (CDI) patients [21,22], with a recent meta-analysis concluding that FMT results in a 90% clinical resolution of CDI symptoms [23]. This is often used in the treatment of gastrointestinal diseases caused by pathogenic microorganisms, which is an important subject due to its relationship with the hypothesis of evolution and improvement in so many other pathologies, such as metabolic syndrome, diabetes, multiple sclerosis, psoriasis, Crohn’s disease, and Parkinson’s disease [20,24]. This may represent a unique approach to patients who would otherwise be prohibited from surgery due to their age, comorbidities, or clinical status and are now a target population for this therapy [24,25].

Initially, it was thought that the donor should be a relative of the patient to have greater efficiency and less chance of rejection. However, recent studies have found that fecal material taken from nonrelatives has the same effect as that from close relatives, so the donor could be a family member, friend, or an unrelated volunteer, as long as they meet the required parameters [22,26,27]. Several scientific studies have highlighted that men and women have different microbial behavior with similar microbiota profiles. Such an issue has indeed been discussed, and considerations about the role of sex hormones in immunity and susceptibility to infections and chronic diseases exist, whereby there are indications that during FMT, gender should be considered [3]. Although this point is still a matter of discussion, it has become clear that microbiota in human and animal models varies according to gender, with males and females having different actions and profiles with the same bacteria [28,29]. In fact, these sex disparities have also been correlated with varying effects on systemic immunology, local GIT inflammation, and susceptibility to various inflammatory illnesses. Indeed, several animal studies have shown that male mice had considerably lower Bacteroidetes levels than females, and a more excellent ratio of Firmicutes and Bacteroidetes than female mice, demonstrating that sex impacts the gut microbiota profile [28,29]. The gut microbiota not only varies between intestinal metabolism and immune reactions (e.g., inflammation) but also presents different profiles between males and females, which could potentially contribute to disparities regarding immunity [30]. Therefore, when considering FMT, studies have already indicated that gender immune differences led to different reactions after FMT procedures. The hypothesis is that this should be regarded as selecting gender-specific gut microbiota composition [30,31], which could open the possibility of developing personalized strategies to target gut microbiota in different diseases.

To ensure that the donor is wholly healthy and to minimize the potential for infectious transmissions of transmissible diseases to the recipient, all FMT donors must undergo rigorous screening [27]. For instance, potential donors are questioned about their travel history, previous surgical interventions, sexual behavior, blood transfusions, and antibiotic use, as well as about their clinical conditions such as asthma, allergies, autoimmune or metabolic diseases, and other factors that increase the risk of transmissible disease [32,33]. In addition, host genetic factors such as diet, innate immune responses, xenobiotic exposure throughout life, and microbial interactions may also shape FMT [34]. Nevertheless, FMT is not a standardized treatment, and protocols differ according to local procedures [26]. Precisely, fecal microbiota administration can be performed in several ways, namely by oral capsules, nasogastric or gastroenteric tube in the upper gastrointestinal tract (UGT), and colonoscopy or enema in the lower gastrointestinal tract (LGT) [7]. Of all these procedures, colonoscopy is the most used method, presenting higher efficacy rates in a global analysis [35] and more advantages than other methods because the fecal substrate is deep in the cecum. In addition, there is no risk of fast removal of the fecal material, which is very likely to happen with enema administrations [3]. Similarly, administering oral capsules has several advantages, such as less invasiveness, higher patient acceptability, and no sedation risk. However, the expensive and enormous capsule burden are the major drawbacks claimed by the patients. Regarding the use of an enema, this can be considered because of its low cost, less invasive method, no associated sedation risk, and the fact that it can be easily repeated. Remarkably, studies have suggested that this method can be used in pediatric patients as well as in multiple administrations of biomaterials to increase the effectiveness of the FMT procedures [36]. Despite promising outcomes, there is no consensus on the ideal route of administration for FMT. Still, Furuya-Kanamori et al. and Nicco et al. suggested that lower gastrointestinal FMT delivery may be preferable to upper gastrointestinal delivery, as patients treated via UGT had 3 times more risk of clinical failure compared to those treated via LGT, despite it being performed in a controlled environment and covering the entire colon [27,37]. Patients’ clinical presentation and personal preferences can help decide on an administration method.

Fresh or frozen stools can be used for the transplant process, as the frozen ones also maintain their molecular integrity and appear not to influence the efficacy outcomes of FMT [26,35,38]. When using fresh material, collecting the biomaterial in a unique disposable container directly at the place where the fecal material will be processed is recommended. Therefore, freshly collected samples must be delivered to the processing site as a ready-to-use product to further increase sample viability and efficacy within all the procedures [39,40]. Before and after the collection of the fecal material, screening must be carried out to ensure that all feces collected and frozen between the two dates are safe [27]. In preparing the donor’s stool for the transplant, some discrepancies exist across labs. However, similar steps are taken, such as combining feces with a bacteriostatic solution, removing particle matter, and increasing the feces delivered to the receiver. In addition, dilution ratios are considered, as they can input possible modifications in the heterogeneity of fecal microorganisms among donors and recipients. So, the sample must be initially diluted, usually with a saline solution or water, followed by homogenization and filtration through a sterile gauze or stainless-steel sieve to remove any particle or gross material, if required. Then the prepared feces can be frozen at least –80°C with the addition of glycerin or used immediately [26,41]. After defrosting, refreezing must not be allowed. More recently, new processing and storage techniques have been introduced. For instance, the fecal aliquot straw technique (FAST) enables a simple and reproducible fecal processing/storage with fewer resources, allowing samples to be stored at extremely low temperatures for lengthy periods and easily subsampled without the need to thaw them several times [24,29].

**Figure 2:**
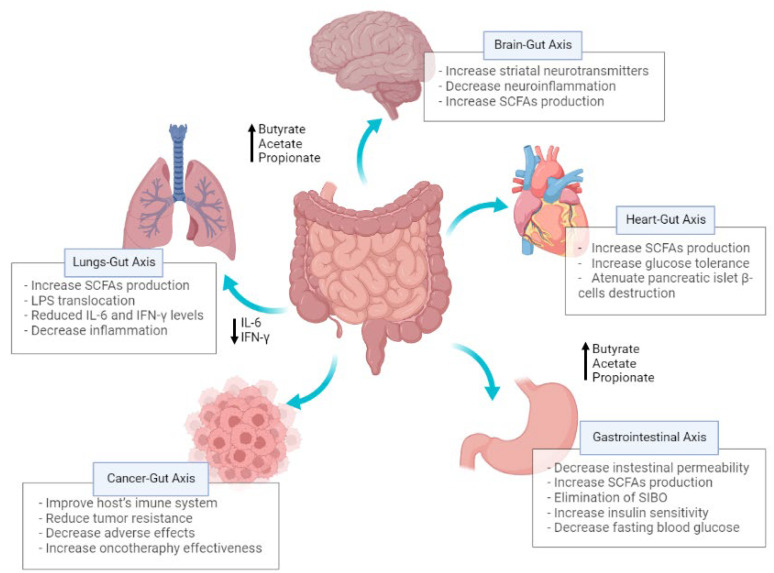
Examining the role of gut microbiota in neurological, respiratory, gastrointestinal, and cardiometabolic disorders and cancer under FMT procedures. From the application point of view, FMT is recognized for reducing intestinal permeability in dysbiotic situations, by increasing the production of butyrate, acetate, and propionate. Such increased levels have been associated with an increase in epithelial barrier integrity and lessened disease severity, especially in brain-gut axis, lungs-gut axis, heart-gut axis, and gastrointestinal axis. In addition, and considering lungs-gut axis in specific, FMT was found to enhance the preservation of alveolar structures and to modulate inflammation through the reduction of IL-6 and IFN- γ levels.

In studies concerning CDI treatment, it has been suggested that only one infusion of FMT is needed to achieve promising results. This is curious and interesting at the same time because *C. difficile* is an opportunistic pathogen that thrives in dysbiotic environments, re-storing healthy gut microbiota and allowing the naturally occurring microbiome to compete with the toxigenic strain of *C. difficile*, thereby leading to the infection’s eventual resolution in 90% of cases. However, for other chronic/inflammatory diseases, such as irritable bowel syndrome and obesity, studies have claimed that more than one transplantation procedure is required since the diversity of pathogenic bacteria involved in the onset and progression of these diseases is much larger than that in CDI. Nevertheless, disease stage and condition cannot be excluded, as variations in different contexts (e.g., inflammation levels, cytokine levels, bacterial levels) may also influence and modulate the treatment requirements [41]. Therefore, several studies prove that FMT is an up-and-coming option for treating various diseases, differing from each other in clinical characteristics and pathophysiology, as shown in Figure 2. Thus, it is expected in the future that we will be able to obtain different FMT protocols for the most varied diseases, achieving greater individualization in the techniques.

## 3. FMT and Neurological Disorders

In the past few years, researchers have found that the gut microbiota affects several parts of the human body, having an essential role in the pathophysiology of certain diseases. It can affect the functioning and development of the central nervous system (CNS) through interaction with the gut–brain axis, affecting the brain’s physiological, behavioral, and cognitive functions [42].

Although studies have reported an alteration in the human gut microbiota composition compared to healthy controls, the exact mechanism of communication between the brain and the gut has not yet been fully understood. Some neurological disorders where this connection is already known to exist are Parkinson’s disease [43], multiple sclerosis [44], autism spectrum disorder [45,46], Alzheimer’s disease [47], depression [48], and others.

This axis is bidirectional crosstalk between the brain and the GIT and its microbiota, involving multiple overlapping pathways such as the endocrine, nervous, and immune systems [49,50]. The bacteria and other microorganisms present in the gut are in constant contact with the intestinal epithelium, which in turn is surrounded by muscles and nerve cells, the so-called enteric nerve system, and these cells are also in connection with the autonomic nervous system, thus providing the complex communication and interaction between the gut and brain [51].

Intestinal dysbiosis can negatively influence the physiology of the gut, causing an inadequate transmission of stimuli along the gut–brain axis and, consequently, causing a change in intestinal permeability and the entry of cytokines into the bloodstream. Lipopolysaccharides (LPSs) are pro-inflammatory endotoxins that can influence the modulation of the central nervous system by modulating the neuropeptide synthesis and increasing the activity of areas connected to emotionalism control [46,50]. This activation of the inflammatory and immune responses has been mentioned as a causal factor for the onset of psychiatric disorders such as depression and schizophrenia [46,49]. Cytokines and metabolites produced by gut microbiota, such as short-chain fatty acids (SCFAs), gamma-aminobutyric acid (GABA), tryptophan, serotonin, and catecholamines, can signal to long-distance targets beyond the GIT through activation of afferent vagal pathways or specific receptors on intestinal cells, or even via an endocrine route [45,49].

Sun et al. showed that when a Parkinson’s disease (PD) mouse model received feces from healthy mice, there was an improvement in motor function, increased striatal neurotransmitters, and decreased neuroinflammation. The opposite was also verified since healthy mice that received feces from PD mice had deteriorated motor function and decreased striatal neurotransmitters compared to controls [52]. With this study, it was evidenced that gut microbiota from PD mice had a decrease in phylum *Firmicutes* and an increase in phylum *Proteobacteria,* which is consistent with observations in human subjects with PD since it is known that increases in *Proteobacteria* can be a consequence of gut inflammation [53]. Huang et al. described a case report in which the PD patient had reduced tremors in both lower limbs and more accessible and quicker defecation after the FMT treatment. Although this was one of the first attempts to treat PD with FMT, the symptoms of shivering and constipation were significantly improved after the transplant. However, other symptoms such as face and neck stiffness showed no significant change, and the improvements demonstrated disappeared over time, indicating that the effect gradually diminished [53]. Consequently, it can be concluded that gut microbiota from PD subjects can induce motor dysfunctions and neurotransmitter loss, and according to several studies, FMT has neuroprotective effects [43,52]. In fact, FMT has attracted the attention of several researchers due to its potential treatment for children with autism spectrum disorder (ASD), and clinical trials are already underway [45]. Kang et al. studied 18 patients diagnosed with ASD, who were re-evaluated two years after FMT, and observed significant improvements in gastrointestinal and behavioral symptoms. After the end of treatment, it was observed that the gastrointestinal benefits obtained with FMT were maintained, and the symptoms of autism improved significantly. These findings demonstrate that FMT is a promising therapy for treating patients with ASD who have gastrointestinal problems [54]. Although the theoretical basis for applying FMT to autism is solid, few tests have been conducted so far, and further investigation is needed [55,56].

Regarding Alzheimer’s disease (AD), several studies have claimed that AD patients have a different gut microbiota composition compared to healthy controls or elderly without dementia [57]. Recently, it has been hypothesized that gut dysbiosis may be associated with AD since it can influence brain activity and cause cognitive dysfunctions [58]. Characterized by the depleted SCFA-producing bacteria such as *Firmicutes* and the enriched inflammation-promoting bacteria such as *Proteobacteria*, the gut microbiota’s role includes direct actions of bacteria, indirect measures, or aging-related processes [42,57]. Studies showed that FMT therapy might be a potential therapeutic strategy for AD by reversing changes in gut microbiota and SCFAs [59].

Although some evidence is available, well-designed large, double-blinded, randomized controlled trials are needed to elucidate the effect of FMT in neurological diseases.

## 4. FMT and Respiratory Diseases

Contrary to what was defended a few years ago, it has been shown that the respiratory tract has its specific microbiome and is colonized by thousands of different microorganisms, although with a much smaller diversity and abundance than the GIT microbiota [60]. Its composition depends on several factors such as inhalation and micro-aspiration of microorganisms and local conditions such as nutrition, temperature, regional oxygenation, and quality and quantity of anti-inflammatory cells [61]. The commensal bacterial species that usually reside on the surfaces of nasal passages interact with the host uninterruptedly, suppressing the colonization of opportunistic pathogens that compete for limited space and nutrients, which are also found in the lower respiratory tract [62,63]. Nevertheless, the lung microbiota communities are likely dynamic since the airways are constantly exposed to air that flows through the upper respiratory tract and oral cavity [64]. Indeed, as these microorganisms correlate with each other in the lungs and several parts of the body, it is not surprising that the imbalance in the gut microbiota has a direct consequence on the development of respiratory diseases; namely, the gut microbiota plays a central role in preserving immunity and in the integrity of internal tissues, where when there is an imbalance, the host’s immune system negatively influences several organs, including the lungs [65]. Nonetheless, although the gut and the lungs are anatomically distinct, they communicate through complex pathways, such as the modulation of immune cells that subsequently migrate into the lung and the translocation of LPSs, reinforcing the idea of an existing gut–lung axis [61]. The respiratory tract microbiota can be influenced by the production of metabolites in the GI tract, such as SCFAs and lipopolysaccharides (LPSs), which can be translocated from the gut to the lung through several channels. Functionally, these can modulate the composition of the respiratory tract and influence the host’s immune response, reinforcing the importance of this axis in these processes [65,66].

Such assumptions in treating gastrointestinal diseases through FMT led us to believe that it can also be used in pathological respiratory conditions such as chronic obstructive pulmonary disease (COPD), pulmonary emphysema, asthma, cystic fibrosis, chronic bronchitis, and lung cancer, in which bacteria play a significant role in perpetuating the inflammation that leads to frequent pulmonary exacerbations. For instance, compared to healthy individuals, patients with asthma or COPD showed increased levels of *Proteobacteria* and *Firmicutes*, while the proportion of *Bacteroidetes* was significantly decreased [63]. In patients with cystic fibrosis, an increase in the *Proteobacteria* phylum and an additional outgrowth of the *Actinobacteria* phylum were reported [13,67]. Lung cancer is one of the deadliest cancers, with a higher incidence and mortality rates, having a higher economic and social burden on the global health systems [64]. The growth of pathogenic bacteria such as *Streptococcus* and *Proteobacteria* has been reported and correlated with high levels of chronic inflammation through the release of inflammatory mediators and metabolites, such as IL-6, IL-1β, IL-12, TNF-α, and LPSs, affecting cell apoptosis and increasing mutations, thereby leading to cellular signaling modifications or direct DNA damages [68]. For instance, in a study performed by Jang et al. [65], the authors addressed whether FMT and a high-fiber diet attenuated emphysema development by suppressing inflammation and apoptosis. From such work, the results demonstrated that alveolar structures were relatively preserved when using fresh feces from mice fed with a high-fiber diet for FMT in emphysema mice. Additionally, they also found that the levels of IL-6 and IFN-γ were reduced, indicating a decrease in inflammation. In addition, the researchers found that when the two variables (FMT and high-fiber diet) were combined, there was a synergistic effect, allowing them to conclude that the combinatory application of FMT and high-fiber diet will lead to better results [69,70].

In another clinical trial, Zhang et al. observed that in patients with spreading respiratory diseases, stool microbial diversity and relative abundance at eight months post-FMT were significantly higher than those before the transplant and were close to the composition of the healthy donors [71]. Additionally, FMT has also demonstrated a potential capability to improve immunotherapy’s efficacy in animal models resistant to treatment [60]. Some clinical trials are in currently progress to address whether FMT may be an adjuvant strategy to increase the effectiveness (and reduce the toxicity) of immunotherapy in lung cancer patients [72]. Such a possibility can lead to the fascinating concept that altering the gut microbiota with FMT may improve the treatment response even in lung cancer resistance to immunotherapy, in which microbiota assessment may be used to identify patients most likely to benefit from this kind of treatment [60,62].

Concerning COVID-19 (for which there is no defined and effective treatment), FMT has gained some evidence in this field, particularly in asymptomatic or mildly symptomatic patients, to be used as adjuvant therapy, namely in patients with associated gastrointestinal manifestations who do not respond to other treatment procedures [73]. Studies in patients with CDI and coexisting COVID-19 concluded that FMT was safe and effective, and both patients experienced mild clinical courses despite having risk factors for COVID-19, such as comorbidities and immunosuppression. One possible explanation is that FMT may mitigate some adverse outcomes through microbiome–immune interactions [74]. However, another concern remains: donor screening is crucial to screen out possible positive cases and prevent transmission of the SARS-CoV-2 virus by using FMT [75]. Thus, clinical and pre-clinical studies should be carried out to investigate the safety and efficacy of FMT in patients infected with SARS-CoV-2 [73].

Altogether, the involvement of the gut microbiota, particularly in respiratory diseases, opens the way for new therapeutic options such as FMT. Nevertheless, although promising results have been achieved, it would be interesting to find out whether FMT from sick donors to healthy hosts could affect the respiratory system, thereby requiring additional pre-clinical and clinical studies to understand the underlying mechanisms of FMT applications.

## 5. FMT and Gastrointestinal Disorders

The gut microbiota is an essential factor in the pathophysiology of many diseases, especially the gastrointestinal tract, contributing to their onset and maintenance [76]. Therefore, modulation of the intestinal microbiota with FMT is expected to restore the correct crosstalk between the host and the microbiome. In fact, the first randomized controlled trials demonstrating FMT efficacy in treating or ameliorating pathological conditions of the GIT started to appear only in recent times [36,77,78]. For instance, *Clostridium difficile* is a Gram-positive anaerobic spore-forming bacteria that produces toxins that cause colon inflammation and is responsible for gastrointestinal illnesses associated with antibiotics, ranging from diarrhea to pseudomembranous colitis, in immunocompromised patients [79]. As demonstrated by Brandt et al. and Quraishi et al., the efficacy of FMT for CDI has success rates of approximately 90%, independent of preparation and route of delivery [23,80].

Irritable bowel syndrome (IBS) is a common gastrointestinal disorder with a significant impact on everyday life [76]. FMT is known for helping reduce intestinal permeability in deregulated gut microbiota by increasing the production of SCFAs, especially butyrate, which will help maintain the integrity of the epithelial barrier and thus decrease the severity of the disease [77,81]. IBS is characterized by chronic alterations in the patient’s microbiota, especially in Firmicutes and Bacteroidetes phyla [5]. Therefore, to overcome dysbiosis, FMT has become a potential alternative therapy for these diseases, with studies highlighting that repeated FMT applications might be required for long-term effects [82,83]. Following this, El-Salhy et al. performed a double-blind, placebo-controlled randomized study with patients with IBS, who were divided into three groups to receive FMT or placebo. FMT was obtained from a healthy donor and administered via the upper gastrointestinal tract. From the results, this study showed a reduction of symptoms three months after FMT, significant improvements in fatigue and quality of life, and mild self-limiting adverse effects. Interestingly, significant changes in the microbiota profile of patients receiving FMT were observed, with higher concentrations of *Eubacterium* and *Lactobacillus* and lower concentrations of Bacteroides [11,84]. In a similar study, Johnsen et al. also evaluated the effect of FMT on IBS-related quality of life and fatigue [85]. They found that FMT induced significant relief on those issues, with lasting results over time, particularly in the subgroups with no excessive functional comorbidity and depression [85]. Nevertheless, establishing ‘super-donors’ remains one of the crucial steps for a successful FMT.

Some forms of inflammatory bowel disease (IBD) manifestation, such as Crohn’s disease (CD) and ulcerative colitis (UC), have been associated with changes in the composition and function of the intestinal microbiota, where there is a loss of enteric bacterial diversity and an increase in specific pathogenic bacteria such as *Enterobacteriaceae* [8]. Preliminary clinical reports of FMT in patients with ulcerative colitis or CD have shown an excellent clinical remission maintained for a long time after FMT. In a systematic review and meta-analysis evaluating the efficacy of FMT as a treatment for patients with IBD, Colman et al. concluded that this procedure is safe but has variable effectiveness [86]. On the other hand, Sun et al. also conducted a systematic review with meta-analysis to determine the efficacy and safety of FMT in ulcerative colitis and demonstrated that FMT is potentially helpful in disease management [87]. Having this in mind, Paramsothy et al. designed a randomized controlled FMT trial for UC patients. They showed that when FMT was applied through colonoscopy and enemas for a specific period, patients who achieved remission showed greater microbial diversity and enrichment in *Eubacterium hallii* and *Roseburia inulivorans* compared to patients who did not achieve remission. In fact, it has been suggested that *E. halli* can increase the production of SCFAs, including butyrate and propionate. The authors also discovered that *Bacteroides* in donor stool samples were associated with remission in patients receiving FMT, and *Streptococcus* species were associated with no response to treatment [88]. On the other hand, hydrogen sulfide, a metabolite originating in the diet, is a toxin associated with the progression of mucosal inflammation in UC by blocking butyrate metabolism in colonic epithelial cells, and a poor detoxification ability may be involved in the pathogenesis of this disease [89,90].

Concerning Crohn’s disease, the effect of FMT has recently been demonstrated to induce remission, and there are currently several clinical trials being performed (NCT04997733 and NCT05321745). In particular, Bak et al. suggested that FMT through the mid-gut may be an option for CD treatment in cases unresponsive to the current conventional therapy (anti-inflammatory agents, steroids, immunosuppressives, and biological medicines), with visible improvements in mucosal lesions, even after ten months of FMT procedure. Interestingly, clinical remission was sustained for more than twelve months [83]. Additionally, it was also found that FMT could eliminate small intestine bacterial overgrowth (SIBO) in most patients with chronic intestinal pseudo-obstruction [91].

## 6. FMT and Cardiometabolic Disorders

Hypertension, hyperlipidemia, hyperglycemia, insulin resistance, and obesity are major risk factors for developing cardiovascular diseases [92]. Meanwhile, it is already known that intestinal dysbiosis is also present in all these clinical conditions, which will increase gut permeability and lead to metabolic endotoxemia and chronic inflammation, thus contributing to metabolic and cardiovascular disease [32].

In cardiovascular diseases, the potential of FMT has been tested in experimental models. In experimental autoimmune myocarditis mouse models, an increase in microbial richness and diversity was found after FMT, with a significant decrease in the Firmicutes/Bacteroidetes ratio being observed [93,94]. Li et al. showed that FMT from hypertensive human donors to germ-free mice could cause elevated blood pressure, describing an exciting and novel causal role of dysbiotic gut microbiota in contributing to the pathogenesis of hypertension, which can represent a direct influence of gut microbiota on blood pressure [95].

Recent studies in animal models and human subjects have revealed that gut dysbiosis is associated with the development of certain metabolic diseases such as obesity, dyslipidemia, type 2 diabetes, and insulin resistance [96]. In addition, changes in the quantitative and qualitative composition of the gut microbiota have shown the ability to inhibit or alter immune responses, thus leading to pro-inflammatory conditions and impaired mucosal barrier function [7,97]. On the other hand, although few studies address this issue, some suggest that FMT from unaffected donors will increase the insulin sensitivity of recipients through regulation of glucagon-like peptide-1 and intestinal gluconeogenesis. This possibility is intriguing since, according to the obtained pre-clinical results, these changes are positively related to an increase in the number of butyrate-producing bacteria and dietary fiber-degrading bacteria [78,98,99,100]. It is known that the translocation of lipopolysaccharides from the intestine to the portal vein is involved in inflammatory conditions associated with obesity and insulin resistance. So, when FMT from lean donors was implemented in an obese mouse model, insulin sensitivity significantly increased because of butyrate-producing bacteria in the intestine, indicating that the gut microbiota could cause and improve obesity and insulin resistance [32]. In obese patients, some studies showed that FMT from lean donors led to minor and transient improvements in glycemic outcomes. However, it is thought that if the procedure were to be repeated several times with the microbiota from lean donors, this would improve metabolic outcomes [97,100]. Yu et al. randomized adults with obesity who were at risk for developing type 2 diabetes to receive either oral FMT capsules from healthy lean donors or a placebo. The results show that oral FMT was safe and tolerable, and possible improvement in metabolism among participants with low microbiome diversity was suggested. Still, and considering type 2 diabetes, in particular, Wang et al. showed that FMT successfully reduced fasting blood glucose levels, improved glucose tolerance in diabetic mice, and attenuated pancreatic islet β-cell destruction, restoring the balance of the gut microbiota and promoting host homeostasis. Their study also demonstrated that the secretion of pro-inflammatory factors decreased, and anti-inflammatory secretion increased in pancreatic tissues [101]. Likewise, in another clinical trial, obese patients receiving FMT from lean donors demonstrated an improvement in insulin sensitivity [102]. Nevertheless, it was also indicated that it seems unlikely that one procedure alone will be sufficient to treat or prevent any disease [103].

So, dietary interventions may be an option for helping maintain FMT efficacy and engraftment in metabolic responses [98].

## 7. FMT and Cancer

In the last few years, the involvement of the gut microbiota in carcinogenesis has been increasingly recognized [100,101]. Efforts have been made to identify which microbial agents are capable of causing it, and it is known that depletion of protective bacteria such as *Lactobacillus* might promote oncogenesis [104]. Modification in gut microbiota composition has been implicated in the development of colorectal cancer, gastric cancer, hepatocellular carcinoma, pancreatic cancer, breast cancer, and melanoma [105]. Indeed, it has been indicated that microorganisms can act directly as cancer-promoting agents through the production of toxic metabolites (vacuolating cytotoxin A and cytotoxin-associated gene A) or some carcinogenic products (TNF-α), or they can act indirectly by inducing inflammation or immunosuppression [106]. Intestinal dysbiosis leads to an SCFA production decrease and exerts pro-inflammatory effects and host DNA damage, which is mediated by microbe-associated molecular patterns (MAMPs) that activate toll-like receptors (TLRs) in macrophages and dendritic cells, thereby promoting the expression of more pro-inflammatory molecules such as IL-23, IL-1, and TNF and inducing local and distant carcinogenesis through the activation of tumorigenic pathways [104,105,107]. Actually, Cui et al. demonstrated that multiple FMT doses increased the survival rate of irradiated mice, improving epithelial integrity and angiogenesis without accelerating tumor growth, suggesting that this method can be used as a new therapeutic approach to improve the prognosis of radiation-induced injuries in cancer by reducing radiotherapy-associated side effects [108,109]. Surprisingly, experimental animal models have shown that before anticancer treatment, FMT can modulate gut microbiota, improve the host’s immune system, and reduce tumor resistance and relevant adverse effects by increasing monotherapy effectiveness [110]. Nonetheless, there are still some concerns regarding FMT applications, namely possible transmissions of pathogens capable of causing viral infections, which would be harmful to immunosuppressed patients, and also the occurrence of diarrhea, cramps, bloating, flatulence, constipation, and low-grade fever in some cases [107,111,112,113,114].

On the other hand, some studies have also shown that the intestinal microbiota can modulate the effectiveness of cancer therapies, especially immunotherapy [105]. Therefore, although FMT may improve the effect of anticancer treatment and reduce the related side effects, additional studies should be considered for a clear assessment of FMT safety alone or in combination with cancer therapies by addressing hypothetical adverse effects and potential underlying mechanisms to overcome them [115]. Indeed, several clinical trials of FMT use in several diseases have been published in the last two years (see Table 1).

## 8. Ethics and Regulation

FMT regulation varies significantly between countries worldwide because several reasons exist that make regulatory bodies undecided on approving fecal microbiota transplantation. Among them are the lack of adequate clinical studies, difficulty in classifying and controlling the human microbiota, and the lack of recognition of FMT as an acceptable therapy by the medical community [131]. Indeed, the execution of this method implies the approach of two ethically controversial areas, namely clinical and therapeutic research and organ transplantation. Additionally, other ethical issues are associated with this method, such as the mode of informed consent and goals that should be thoroughly explained to participants before obtaining their verbal consent, privacy protection of any clinical information of each participant, and ownership of samples. However, informed consent may be difficult due to the vulnerability of patients, the untested nature of the method, and the lack of information on potential side effects. Moreover, patients’ participation must be voluntary under clinical trials, and anonymity must be guaranteed [131]. Despite all these considerations, Europe uses FMT with high standards to provide safe approaches. Although the European Medicines Agency (EMA) has not yet taken any position on the classification of the FMT, this procedure is considered an experimental method performed only in patients in research environments. Nevertheless, a significant gap in FMT coverage demonstrates a need to raise awareness among the medical community and the general population, increasing the possibility of people being more receptive to this method [132,133]. Contrary to the EMA, the Food and Drug Administration (FDA) considers FMT a new experimental drug, requiring scientists and doctors to use the treatment. For instance, it has been described that FMT can be used to treat patients with *C. difficile* infection not responsive to standard therapies under the agency’s policy of application. In fact, FDA does not restrict the use of FMT to any particular route of administration such as colonoscopy, enema, or oral capsules. Therefore, FMT presents a new regulatory challenge and provides a chance for critical thinking about the most appropriate ways to oversee this new technology [134]. Notably, in Canada, FMT is approved for clinical use in patients with recurrent *C. difficile* infection, thereby leading to clinical benefits. Even though patients should meet specific clinical criteria to receive this treatment, due to its clinical contraindications, the inability to find a suitable donor and choose the correct administration method is often a reason to consider this treatment as an option [7,23]. However, patients with other conditions, such as those referred to in this article, may be able to access this treatment through participation in clinical trials.

Therefore, the ethical and legal implications of FMT are worthy of careful consideration by healthcare and regulatory professionals, and technical and public discussions about this should be considered.

## 9. Conclusions and Future Perspectives

The knowledge of the role of gut microbiota in health and disease has enormously grown in the past decades. A deep relationship between this system and almost all human infections has been found by considering this a potential source of disease understanding and therapeutical developments.

Studies have shown that FMT is a new and promising method for treatment in clinical situations characterized by the development of antibacterial resistance and changes in the composition of the intestinal microbiota, as well as in the presence of several other diseases described throughout this review. Despite a large amount of data, clinical studies, and recommendations, there are still many doubts about its implementation. In addition, although this procedure is being described as safe, the ‘temporary’ side effects (e.g., diarrhea, cramps or abdominal pain, increased frequency of bowel movements, low-grade fever, bloating, flatulence, and constipation) cannot be discredited. In fact, there are insufficient long-term follow-up data, so infection, inflammation, or gastrointestinal malignancies must be considered as potential future problems, requiring further investigation. Therefore, the characterization of super-donors should be considered, as it will allow the development of more specific FMT formulations to help standardize therapy and reduce variability in patients’ responses. Actually, if rules for donor selection can be systematized, further investigation should be performed to address issues related to the frequency of donor screening, biomaterial processing, and storage time. It will also be quite challenging to understand the impact of (bio)engineering advances in the processing of FMT and how different preconditional (environmental) conditions might be a tool to enhance the feasibility, safety, and disease-specific therapeutical potential of FMT for clinical use.

## Figures and Tables

**Table 1:** Fecal microbiota transplantation tested in clinical trials in several diseases.

Study	Year	Disease	Treatment	Type of study	Via	Target	Outcome	Reference
Baruch En et al.	2021	Melanoma	FMT	CT	Colonoscopy and oral capsules	Reinduction of anti-PD-1 immunotherapy	Favorable changes in immune cell infiltrates and gene expression profiles in both the gut lamina propria and the tumor microenvironment. Safe and feasible.	[116]
Diwakar Davar et al.	2021	Melanoma	FMT	CT	Colonoscopy	Resistance to anti-PD-1	Increased abundance of anti-PD-1 response; increased CD8+ T cell activation; decreased frequency of IL-8-expressing myeloid cells; distinct proteomic and metabolomic signatures.	[117]
Holvoet T et al.	2021	IBS	FMT	CT	Nasojejunal tube	Microbial ecosystem	FMT relieved symptoms compared with placebo, although the effects decreased over 1 year.	[118]
Pieter de Groot et al.	2021	T1DM	FMT	CT	Nasoduodenal tube	Preservation of stimulated C peptide release	FMT halts decline in endogenous insulin production in T1DM patients 12 months after disease onset.	[119]
Karjalainen EK et al.	2021	UC	FMT	CT	Endoscopy and transanal catheter	Efficacy and safety of FMT in chronic pouchitis	FMT was not effective in the treatment of chronic pouchitis. The safety profile was good.	[120]
Gianluca Ianiro et al.	2021	IBS and recurrent CDI	FMT	CT	Colonoscopy	Microbial ecosystem	FMT appears to be highly effective and safe and not only eradicates CDI but also improves IBD activity.	[121]
Valentim Mocanu et al.	2021	Obesity and metabolic syndrome	FMT	CT	Oral capsules	Microbial ecosystem	Single-dose oral FMT combined with daily low-fermentable fiber supplementation improves insulin sensitivity.	[122]
El-Salhy M et al.	2021	IBS	FMT	CT	Nasoduodenal tube	SCFA levels	FMT increases fecal SCFA levels in IBS patients. The increase in the butyric acid level is inversely correlated with symptoms in IBS patients following FMT.	[123]
Segal A et al.	2021	PD	FMT	CT	Colonoscopy	Motor and non-motor symptoms in PD patients	FMT improves PD motor and non-motor symptoms, including constipation. Good safety profile.	[124]
Wu LH et al.	2021	COVID-19	FMT	CT	Nasojejunal tube	Intestinal mucosal barrier function	Improvement of intestinal mucosal barrier function, inflammatory response, and immunity. FMT is efficacious and safe.	[125]
Dafa Ding et al.	2022	T2DM	FMT	CT	Transendoscopic enteral tubing (TET)	Glucose homeostasis	T2DM patients can potentially benefit from FMT. Pretreated abundance of *Rikenellaceae* and *Anaerotruncus* may serve as potential biomarkers for selecting T2DM patients to receive FMT.	[126]
Huang C et al.	2022	UC	FMT	CT	Endoscopic spray and retention enema	Microbial ecosystem	FMT therapy was as effective as glucocorticoids to induce remission in active mild to moderate UC, accompanied by fewer adverse events.	[127]
Su L et al.	2022	T2DM	FMT	CT	Oral capsules	Microbial ecosystem	FMT worked in conjunction with dietary intervention to accelerate the weight loss effect	[128]
Haifer C et al.	2022	UC	FMT	CT	Oral capsules	Corticosteroid-free clinical remission with endoscopic remission	FMT induced remission in patients with active ulcerative colitis. Continuing FMT was well tolerated and appeared to demonstrate clinical, endoscopic, and histological efficacy.	[129]
Mazzawi T et al.	2022	IBS	FMT	CT	Colonoscopy	Colonic enteroendocrine (CEE) cell densities	CEE cell densities significantly change after FMT	[130]

Legend: IBS, irritable bowel syndrome; CDI, *Clostridium difficile* infection; CT, clinical trial; FMT, fecal microbiota transplantation; CEE, colon enteroendocrine; SCFA, short-chain fatty acid; UC, ulcerative colitis; IBD, inflammatory bowel disease; PD-1, programmed cell death protein 1; PD, Parkinson’s disease; T1DM, type 1 diabetes mellitus; T2DM, type 2 diabetes mellitus.

## Data Availability

Not applicable.

## References

[1] Rinninella E., Raoul P., Cintoni M., Franceschi F., Miggiano G.A.D., Gasbarrini A., Mele M.C. (2019). What is the Healthy Gut Microbiota Composition? A Changing Ecosystem across Age, Environment, Diet, and Diseases. Microorganisms.

[2] Fan Y., Pedersen O. (2021). Gut microbiota in human metabolic health and disease. Nat. Rev. Microbiol..

[3] Antushevich H. (2020). Fecal microbiota transplantation in disease therapy. Clin. Chim. Acta Int. J. Clin. Chem..

[4] The Human Microbiome Project Consortium (2012). Structure, function and diversity of the healthy human microbiome. Nature.

[5] Glassner K.L., Abraham B.P., Quigley E.M.M. (2020). The microbiome and inflammatory bowel disease. J. Allergy Clin. Immunol..

[6] Tamburini S., Shen N., Wu H.C., Clemente J.C. (2016). The microbiome in early life: Implications for health outcomes. Nat. Med..

[7] Ooijevaar R.E., Terveer E.M., Verspaget H.W., Kuijper E.J., Keller J.J. (2019). Clinical Application and Potential of Fecal Microbiota Transplantation. Annu. Rev. Med..

[8] Durack J., Lynch S.V. (2019). The gut microbiome: Relationships with disease and opportunities for therapy. J. Exp. Med..

[9] Durand G.A., Raoult D., Dubourg G. (2019). Antibiotic discovery: History, methods and perspectives. Int. J. Antimicrob. Agents.

[10] Rothschild D., Weissbrod O., Barkan E., Kurilshikov A., Korem T., Zeevi D., Costea P.I., Godneva A., Kalka I.N., Bar N. (2018). Environment dominates over host genetics in shaping human gut microbiota. Nature.

[11] Singh R., Zogg H., Wei L., Bartlett A., Ghoshal U.C., Rajender S., Ro S. (2021). Gut Microbial Dysbiosis in the Pathogenesis of Gastrointestinal Dysmotility and Metabolic Disorders. J. Neurogastroenterol. Motil..

[12] Knauf F., Brewer J.R., Flavell R.A. (2019). Immunity, microbiota and kidney disease. Nat. Rev. Nephrol..

[13] Marsland B.J., Trompette A., Gollwitzer E.S. (2015). The Gut-Lung Axis in Respiratory Disease. Ann. Am. Thorac. Soc..

[14] Dominguez-Bello M.G., Godoy-Vitorino F., Knight R., Blaser M.J. (2019). Role of the microbiome in human development. Gut.

[15] Salvucci E. (2019). The human-microbiome superorganism and its modulation to restore health. Int. J. Food Sci. Nutr..

[16] Quigley E.M. (2013). Gut bacteria in health and disease. Gastroenterol. Hepatol..

[17] Thursby E., Juge N. (2017). Introduction to the human gut microbiota. Biochem. J..

[18] Kho Z.Y., Lal S.K. (2018). The Human Gut Microbiome—A Potential Controller of Wellness and Disease. Front. Microbiol..

[19] Cabrera-Perez J., Badovinac V.P., Griffith T.S. (2017). Enteric immunity, the gut microbiome, and sepsis: Rethinking the germ theory of disease. Exp. Biol. Med..

[20] Leshem A., Horesh N., Elinav E. (2019). Fecal Microbial Transplantation and Its Potential Application in Cardiometabolic Syndrome. Front. Immunol..

[21] Czepiel J., Drozdz M., Pituch H., Kuijper E.J., Perucki W., Mielimonka A., Goldman S., Wultanska D., Garlicki A., Biesiada G. (2019). Clostridium difficile infection: Review. Eur. J. Clin. Microbiol. Infect. Dis. Off. Publ. Eur. Soc. Clin. Microbiol..

[22] Lynch S.M., Mu J., Grady J.J., Stevens R.G., Devers T.J. (2019). Fecal Microbiota Transplantation for Clostridium difficile Infection: A One-Center Experience. Dig. Dis..

[23] Quraishi M.N., Widlak M., Bhala N., Moore D., Price M., Sharma N., Iqbal T.H. (2017). Systematic review with meta-analysis: The efficacy of faecal microbiota transplantation for the treatment of recurrent and refractory Clostridium difficile infection. Aliment. Pharmacol. Ther..

[24] Cheng Y.W., Phelps E., Nemes S., Rogers N., Sagi S., Bohm M., El-Halabi M., Allegretti J.R., Kassam Z., Xu H. (2020). Fecal Microbiota Transplant Decreases Mortality in Patients with Refractory Severe or Fulminant Clostridioides difficile Infection. Clin. Gastroenterol. Hepatol. Off. Clin. Pract. J. Am. Gastroenterol. Assoc..

[25] Gweon T.G., Na S.Y. (2021). Next Generation Fecal Microbiota Transplantation. Clin. Endosc..

[26] Liubakka A., Vaughn B.P. (2016). Clostridium difficile Infection and Fecal Microbiota Transplant. AACN Adv. Crit. Care.

[27] Nicco C., Paule A., Konturek P., Edeas M. (2020). From Donor to Patient: Collection, Preparation and Cryopreservation of Fecal Samples for Fecal Microbiota Transplantation. Diseases.

[28] Wang J., Zhong Y., Zhu H., Mahgoub O.K., Jian Z., Gu L., Xiong X. (2022). Different gender-derived gut microbiota influence stroke outcomes by mitigating inflammation. J. Neuroinflamm..

[29] Vemuri R., Sylvia K.E., Klein S.L., Forster S.C., Plebanski M., Eri R., Flanagan K.L. (2019). The microgenderome revealed: Sex differences in bidirectional interactions between the microbiota, hormones, immunity and disease susceptibility. Semin. Immunopathol..

[30] Fransen F., van Beek A.A., Borghuis T., Meijer B., Hugenholtz F., van der Gaast-de Jongh C., Savelkoul H.F., de Jonge M.I., Faas M.M., Boekschoten M.V. (2017). The Impact of Gut Microbiota on Gender-Specific Differences in Immunity. Front. Immunol..

[31] Kim Y.S., Unno T., Kim B.Y., Park M.S. (2020). Sex Differences in Gut Microbiota. World J. Mens Health.

[32] Smits L.P., Bouter K.E., de Vos W.M., Borody T.J., Nieuwdorp M. (2013). Therapeutic potential of fecal microbiota transplantation. Gastroenterology.

[33] McSweeney B., Allegretti J.R., Fischer M., Xu H., Goodman K.J., Monaghan T., McLeod C., Mullish B.H., Petrof E.O., Phelps E.L. (2020). In search of stool donors: A multicenter study of prior knowledge, perceptions, motivators, and deterrents among potential donors for fecal microbiota transplantation. Gut Microbes.

[34] Ng S.C., Kamm M.A., Yeoh Y.K., Chan P.K.S., Zuo T., Tang W., Sood A., Andoh A., Ohmiya N., Zhou Y. (2020). Scientific frontiers in faecal microbiota transplantation: Joint document of Asia-Pacific Association of Gastroenterology (APAGE) and Asia-Pacific Society for Digestive Endoscopy (APSDE). Gut.

[35] Ianiro G., Maida M., Burisch J., Simonelli C., Hold G., Ventimiglia M., Gasbarrini A., Cammarota G. (2018). Efficacy of different faecal microbiota transplantation protocols for Clostridium difficile infection: A systematic review and meta-analysis. United Eur. Gastroenterol. J..

[36] Vindigni S.M., Surawicz C.M. (2017). Fecal Microbiota Transplantation. Gastroenterol. Clin. N. Am..

[37] Furuya-Kanamori L., Doi S.A., Paterson D.L., Helms S.K., Yakob L., McKenzie S.J., Garborg K., Emanuelsson F., Stollman N., Kronman M.P. (2017). Upper Versus Lower Gastrointestinal Delivery for Transplantation of Fecal Microbiota in Recurrent or Refractory Clostridium difficile Infection: A Collaborative Analysis of Individual Patient Data From 14 Studies. J. Clin. Gastroenterol..

[38] Kumar V., Fischer M. (2020). Expert opinion on fecal microbiota transplantation for the treatment of Clostridioides difficile infection and beyond. Expert Opin. Biol. Ther..

[39] Bakken J.S., Borody T., Brandt L.J., Brill J.V., Demarco D.C., Franzos M.A., Kelly C., Khoruts A., Louie T., Martinelli L.P. (2011). Treating Clostridium difficile infection with fecal microbiota transplantation. Clin. Gastroenterol. Hepatol. Off. Clin. Pract. J. Am. Gastroenterol. Assoc..

[40] Lee C.H., Steiner T., Petrof E.O., Smieja M., Roscoe D., Nematallah A., Weese J.S., Collins S., Moayyedi P., Crowther M. (2016). Frozen vs Fresh Fecal Microbiota Transplantation and Clinical Resolution of Diarrhea in Patients With Recurrent Clostridium difficile Infection: A Randomized Clinical Trial. JAMA.

[41] Cammarota G., Ianiro G., Bibbo S., Gasbarrini A. (2014). Gut microbiota modulation: Probiotics, antibiotics or fecal microbiota transplantation?. Intern. Emerg. Med..

[42] Vendrik K.E.W., Ooijevaar R.E., de Jong P.R.C., Laman J.D., van Oosten B.W., van Hilten J.J., Ducarmon Q.R., Keller J.J., Kuijper E.J., Contarino M.F. (2020). Fecal Microbiota Transplantation in Neurological Disorders. Front. Cell. Infect. Microbiol..

[43] Unger M.M., Spiegel J., Dillmann K.U., Grundmann D., Philippeit H., Burmann J., Fassbender K., Schwiertz A., Schafer K.H. (2016). Short chain fatty acids and gut microbiota differ between patients with Parkinson’s disease and age-matched controls. Parkinsonism Relat. Disord..

[44] Chen J., Chia N., Kalari K.R., Yao J.Z., Novotna M., Paz Soldan M.M., Luckey D.H., Marietta E.V., Jeraldo P.R., Chen X. (2016). Multiple sclerosis patients have a distinct gut microbiota compared to healthy controls. Sci. Rep..

[45] Garcia-Gutierrez E., Narbad A., Rodriguez J.M. (2020). Autism Spectrum Disorder Associated With Gut Microbiota at Immune, Metabolomic, and Neuroactive Level. Front. Neurosci..

[46] Mangiola F., Ianiro G., Franceschi F., Fagiuoli S., Gasbarrini G., Gasbarrini A. (2016). Gut microbiota in autism and mood disorders. World J. Gastroenterol..

[47] Haran J.P., Bhattarai S.K., Foley S.E., Dutta P., Ward D.V., Bucci V., McCormick B.A. (2019). Alzheimer’s Disease Microbiome Is Associated with Dysregulation of the Anti-Inflammatory P-Glycoprotein Pathway. mBio.

[48] Limbana T., Khan F., Eskander N. (2020). Gut Microbiome and Depression: How Microbes Affect the Way We Think. Cureus.

[49] Arneth B.M. (2018). Gut-brain axis biochemical signalling from the gastrointestinal tract to the central nervous system: Gut dysbiosis and altered brain function. Postgrad. Med. J..

[50] Wang H.X., Wang Y.P. (2016). Gut Microbiota-brain Axis. Chin. Med. J..

[51] Mayer E.A., Savidge T., Shulman R.J. (2014). Brain-gut microbiome interactions and functional bowel disorders. Gastroenterology.

[52] Sun M.F., Zhu Y.L., Zhou Z.L., Jia X.B., Xu Y.D., Yang Q., Cui C., Shen Y.Q. (2018). Neuroprotective effects of fecal microbiota transplantation on MPTP-induced Parkinson’s disease mice: Gut microbiota, glial reaction and TLR4/TNF-alpha signaling pathway. Brain Behav. Immun..

[53] Huang H., Xu H., Luo Q., He J., Li M., Chen H., Tang W., Nie Y., Zhou Y. (2019). Fecal microbiota transplantation to treat Parkinson’s disease with constipation: A case report. Medicine.

[54] Kang D.W., Adams J.B., Coleman D.M., Pollard E.L., Maldonado J., McDonough-Means S., Caporaso J.G., Krajmalnik-Brown R. (2019). Long-term benefit of Microbiota Transfer Therapy on autism symptoms and gut microbiota. Sci. Rep..

[55] Bested A.C., Logan A.C., Selhub E.M. (2013). Intestinal microbiota, probiotics and mental health: From Metchnikoff to modern advances: Part I - autointoxication revisited. Gut Pathog..

[56] Aroniadis O.C., Brandt L.J. (2013). Fecal microbiota transplantation: Past, present and future. Curr. Opin. Gastroenterol..

[57] Liu P., Wu L., Peng G., Han Y., Tang R., Ge J., Zhang L., Jia L., Yue S., Zhou K. (2019). Altered microbiomes distinguish Alzheimer’s disease from amnestic mild cognitive impairment and health in a Chinese cohort. Brain Behav. Immun..

[58] Angelucci F., Cechova K., Amlerova J., Hort J. (2019). Antibiotics, gut microbiota, and Alzheimer’s disease. J. Neuroinflammation.

[59] Zhan G., Yang N., Li S., Huang N., Fang X., Zhang J., Zhu B., Yang L., Yang C., Luo A. (2018). Abnormal gut microbiota composition contributes to cognitive dysfunction in SAMP8 mice. Aging.

[60] Mathieu E., Escribano-Vazquez U., Descamps D., Cherbuy C., Langella P., Riffault S., Remot A., Thomas M. (2018). Paradigms of Lung Microbiota Functions in Health and Disease, Particularly, in Asthma. Front. Physiol..

[61] Enaud R., Prevel R., Ciarlo E., Beaufils F., Wieers G., Guery B., Delhaes L. (2020). The Gut-Lung Axis in Health and Respiratory Diseases: A Place for Inter-Organ and Inter-Kingdom Crosstalks. Front. Cell. Infect. Microbiol..

[62] Hanada S., Pirzadeh M., Carver K.Y., Deng J.C. (2018). Respiratory Viral Infection-Induced Microbiome Alterations and Secondary Bacterial Pneumonia. Front. Immunol..

[63] Brugger S.D., Bomar L., Lemon K.P. (2016). Commensal-Pathogen Interactions along the Human Nasal Passages. PLoS Pathog..

[64] Ramirez-Labrada A.G., Isla D., Artal A., Arias M., Rezusta A., Pardo J., Galvez E.M. (2020). The Influence of Lung Microbiota on Lung Carcinogenesis, Immunity, and Immunotherapy. Trends Cancer.

[65] Wu B.G., Segal L.N. (2018). The Lung Microbiome and Its Role in Pneumonia. Clin. Chest Med..

[66] Dang A.T., Marsland B.J. (2019). Microbes, metabolites, and the gut-lung axis. Mucosal Immunol..

[67] Sze M.A., Dimitriu P.A., Hayashi S., Elliott W.M., McDonough J.E., Gosselink J.V., Cooper J., Sin D.D., Mohn W.W., Hogg J.C. (2012). The lung tissue microbiome in chronic obstructive pulmonary disease. Am. J. Respir. Crit. Care Med..

[68] Xu N., Wang L., Li C., Ding C., Li C., Fan W., Cheng C., Gu B. (2020). Microbiota dysbiosis in lung cancer: Evidence of association and potential mechanisms. Transl. Lung Cancer Res..

[69] Jang Y.O., Lee S.H., Choi J.J., Kim D.H., Choi J.M., Kang M.J., Oh Y.M., Park Y.J., Shin Y., Lee S.W. (2020). Fecal microbial transplantation and a high fiber diet attenuates emphysema development by suppressing inflammation and apoptosis. Exp. Mol. Med..

[70] Kang Y., Cai Y. (2018). Future prospect of faecal microbiota transplantation as a potential therapy in asthma. Allergol. Immunopathol..

[71] Zhang T., Ding X., Dai M., Zhang H., Xiao F., He X., Zhang F., Zhang X. (2021). Washed microbiota transplantation in patients with respiratory spreading diseases: Practice recommendations. Med. Microecol..

[72] Liu X., Cheng Y., Zang D., Zhang M., Li X., Liu D., Gao B., Zhou H., Sun J., Han X. (2021). The Role of Gut Microbiota in Lung Cancer: From Carcinogenesis to Immunotherapy. Front. Oncol..

[73] Nejadghaderi S.A., Nazemalhosseini-Mojarad E., Asadzadeh Aghdaei H. (2021). Fecal microbiota transplantation for COVID-19; a potential emerging treatment strategy. Med. Hypotheses.

[74] Biliński J., Winter K., Jasiński M., Szczęś A., Bilinska N., Mullish B.H., Małecka-Panas E., Basak G.W. (2022). Rapid resolution of COVID-19 after faecal microbiota transplantation. Gut.

[75] Green C.A., Quraishi M.N., Shabir S., Sharma N., Hansen R., Gaya D.R., Hart A.L., Loman N.J., Iqbal T.H. (2020). Screening faecal microbiota transplant donors for SARS-CoV-2 by molecular testing of stool is the safest way forward. Lancet Gastroenterol. Hepatol..

[76] Benech N., Sokol H. (2020). Fecal microbiota transplantation in gastrointestinal disorders: Time for precision medicine. Genome Med..

[77] Manichanh C., Borruel N., Casellas F., Guarner F. (2012). The gut microbiota in IBD. Nat. Rev. Gastroenterol. Hepatol..

[78] de Groot P.F., Frissen M.N., de Clercq N.C., Nieuwdorp M. (2017). Fecal microbiota transplantation in metabolic syndrome: History, present and future. Gut Microbes.

[79] Matsuoka K., Mizuno S., Hayashi A., Hisamatsu T., Naganuma M., Kanai T. (2014). Fecal microbiota transplantation for gastrointestinal diseases. Keio J. Med..

[80] Brandt L.J., Aroniadis O.C., Mellow M., Kanatzar A., Kelly C., Park T., Stollman N., Rohlke F., Surawicz C. (2012). Long-term follow-up of colonoscopic fecal microbiota transplant for recurrent Clostridium difficile infection. Am. J. Gastroenterol..

[81] Sunkara T., Rawla P., Ofosu A., Gaduputi V. (2018). Fecal microbiota transplant—A new frontier in inflammatory bowel disease. J. Inflamm. Res..

[82] Aroniadis O.C., Brandt L.J. (2014). Intestinal microbiota and the efficacy of fecal microbiota transplantation in gastrointestinal disease. Gastroenterol. Hepatol..

[83] Bak S.H., Choi H.H., Lee J., Kim M.H., Lee Y.H., Kim J.S., Cho Y.S. (2017). Fecal microbiota transplantation for refractory Crohn’s disease. Intest. Res..

[84] El-Salhy M., Hatlebakk J.G., Gilja O.H., Brathen Kristoffersen A., Hausken T. (2020). Efficacy of faecal microbiota transplantation for patients with irritable bowel syndrome in a randomised, double-blind, placebo-controlled study. Gut.

[85] Johnsen P.H., Hilpusch F., Valle P.C., Goll R. (2020). The effect of fecal microbiota transplantation on IBS related quality of life and fatigue in moderate to severe non-constipated irritable bowel: Secondary endpoints of a double blind, randomized, placebo-controlled trial. EBioMedicine.

[86] Colman R.J., Rubin D.T. (2014). Fecal microbiota transplantation as therapy for inflammatory bowel disease: A systematic review and meta-analysis. J. Crohn’s Colitis.

[87] Sun D., Li W., Li S., Cen Y., Xu Q., Li Y., Sun Y., Qi Y., Lin Y., Yang T. (2016). Fecal Microbiota Transplantation as a Novel Therapy for Ulcerative Colitis: A Systematic Review and Meta-Analysis. Medicine.

[88] Paramsothy S., Nielsen S., Kamm M.A., Deshpande N.P., Faith J.J., Clemente J.C., Paramsothy R., Walsh A.J., van den Bogaerde J., Samuel D. (2019). Specific Bacteria and Metabolites Associated with Response to Fecal Microbiota Transplantation in Patients with Ulcerative Colitis. Gastroenterology.

[89] Teigen L.M., Geng Z., Sadowsky M.J., Vaughn B.P., Hamilton M.J., Khoruts A. (2019). Dietary Factors in Sulfur Metabolism and Pathogenesis of Ulcerative Colitis. Nutrients.

[90] Mishima Y., Sartor R.B. (2020). Manipulating resident microbiota to enhance regulatory immune function to treat inflammatory bowel diseases. J. Gastroenterol..

[91] Gu L., Ding C., Tian H., Yang B., Zhang X., Hua Y., Zhu Y., Gong J., Zhu W., Li J. (2017). Serial Frozen Fecal Microbiota Transplantation in the Treatment of Chronic Intestinal Pseudo-obstruction: A Preliminary Study. J. Neurogastroenterol. Motil..

[92] Singh V., Yeoh B.S., Vijay-Kumar M. (2016). Gut microbiome as a novel cardiovascular therapeutic target. Curr. Opin. Pharmacol..

[93] Liu Y., Zhang F.M., Hu W.Z. (2020). Hypertension: Microbiota-targeting treatment. Chin. Med. J..

[94] Hu X.F., Zhang W.Y., Wen Q., Chen W.J., Wang Z.M., Chen J., Zhu F., Liu K., Cheng L.X., Yang J. (2019). Fecal microbiota transplantation alleviates myocardial damage in myocarditis by restoring the microbiota composition. Pharmacol. Res..

[95] Li J., Zhao F., Wang Y., Chen J., Tao J., Tian G., Wu S., Liu W., Cui Q., Geng B. (2017). Gut microbiota dysbiosis contributes to the development of hypertension. Microbiome.

[96] Moszak M., Szulinska M., Bogdanski P. (2020). You Are What You Eat-The Relationship between Diet, Microbiota, and Metabolic Disorders-A Review. Nutrients.

[97] Lee P., Yacyshyn B.R., Yacyshyn M.B. (2019). Gut microbiota and obesity: An opportunity to alter obesity through faecal microbiota transplant (FMT). Diabetes Obes. Metab..

[98] Zhang Z., Mocanu V., Cai C., Dang J., Slater L., Deehan E.C., Walter J., Madsen K.L. (2019). Impact of Fecal Microbiota Transplantation on Obesity and Metabolic Syndrome-A Systematic Review. Nutrients.

[99] Smits L.P., Kootte R.S., Levin E., Prodan A., Fuentes S., Zoetendal E.G., Wang Z., Levison B.S., Cleophas M.C.P., Kemper E.M. (2018). Effect of Vegan Fecal Microbiota Transplantation on Carnitine- and Choline-Derived Trimethylamine-N-Oxide Production and Vascular Inflammation in Patients With Metabolic Syndrome. J. Am. Heart Assoc..

[100] Kootte R.S., Levin E., Salojarvi J., Smits L.P., Hartstra A.V., Udayappan S.D., Hermes G., Bouter K.E., Koopen A.M., Holst J.J. (2017). Improvement of Insulin Sensitivity after Lean Donor Feces in Metabolic Syndrome Is Driven by Baseline Intestinal Microbiota Composition. Cell Metab..

[101] Wang H., Lu Y., Yan Y., Tian S., Zheng D., Leng D., Wang C., Jiao J., Wang Z., Bai Y. (2019). Promising Treatment for Type 2 Diabetes: Fecal Microbiota Transplantation Reverses Insulin Resistance and Impaired Islets. Front. Cell. Infect. Microbiol..

[102] Aron-Wisnewsky J., Clement K., Nieuwdorp M. (2019). Fecal Microbiota Transplantation: A Future Therapeutic Option for Obesity/Diabetes?. Curr. Diabetes Rep..

[103] Yu E.W., Gao L., Stastka P., Cheney M.C., Mahabamunuge J., Torres Soto M., Ford C.B., Bryant J.A., Henn M.R., Hohmann E.L. (2020). Fecal microbiota transplantation for the improvement of metabolism in obesity: The FMT-TRIM double-blind placebo-controlled pilot trial. PLoS Med..

[104] Lam S.Y., Yu J., Wong S.H., Peppelenbosch M.P., Fuhler G.M. (2017). The gastrointestinal microbiota and its role in oncogenesis. Best Pract. Res. Clin. Gastroenterol..

[105] Chen D., Wu J., Jin D., Wang B., Cao H. (2019). Fecal microbiota transplantation in cancer management: Current status and perspectives. Int. J. Cancer.

[106] Zitvogel L., Ma Y., Raoult D., Kroemer G., Gajewski T.F. (2018). The microbiome in cancer immunotherapy: Diagnostic tools and therapeutic strategies. Science.

[107] Yu L.X., Schwabe R.F. (2017). The gut microbiome and liver cancer: Mechanisms and clinical translation. Nat. Rev. Gastroenterol. Hepatol..

[108] Cui M., Xiao H., Li Y., Zhou L., Zhao S., Luo D., Zheng Q., Dong J., Zhao Y., Zhang X. (2017). Faecal microbiota transplantation protects against radiation-induced toxicity. EMBO Mol. Med..

[109] Wardill H.R., Secombe K.R., Bryant R.V., Hazenberg M.D., Costello S.P. (2019). Adjunctive fecal microbiota transplantation in supportive oncology: Emerging indications and considerations in immunocompromised patients. EBioMedicine.

[110] Wu X., Zhang T., Chen X., Ji G., Zhang F. (2019). Microbiota transplantation: Targeting cancer treatment. Cancer Lett..

[111] Dang X., Xu M., Liu D., Zhou D., Yang W. (2020). Assessing the efficacy and safety of fecal microbiota transplantation and probiotic VSL#3 for active ulcerative colitis: A systematic review and meta-analysis. PLoS ONE.

[112] Wang S., Xu M., Wang W., Cao X., Piao M., Khan S., Yan F., Cao H., Wang B. (2016). Systematic Review: Adverse Events of Fecal Microbiota Transplantation. PLoS ONE.

[113] Park S.Y., Seo G.S. (2021). Fecal Microbiota Transplantation: Is It Safe?. Clin. Endosc..

[114] Caldeira L.F., Borba H.H., Tonin F.S., Wiens A., Fernandez-Llimos F., Pontarolo R. (2020). Fecal microbiota transplantation in inflammatory bowel disease patients: A systematic review and meta-analysis. PLoS ONE.

[115] Kelly C.R., Kahn S., Kashyap P., Laine L., Rubin D., Atreja A., Moore T., Wu G. (2015). Update on Fecal Microbiota Transplantation 2015: Indications, Methodologies, Mechanisms, and Outlook. Gastroenterology.

[116] Baruch E.N., Youngster I., Ben-Betzalel G., Ortenberg R., Lahat A., Katz L., Adler K., Dick-Necula D., Raskin S., Bloch N. (2021). Fecal microbiota transplant promotes response in immunotherapy-refractory melanoma patients. Science.

[117] Davar D., Dzutsev A.K., McCulloch J.A., Rodrigues R.R., Chauvin J.M., Morrison R.M., Deblasio R.N., Menna C., Ding Q., Pagliano O. (2021). Fecal microbiota transplant overcomes resistance to anti-PD-1 therapy in melanoma patients. Science.

[118] Holvoet T., Joossens M., Vazquez-Castellanos J.F., Christiaens E., Heyerick L., Boelens J., Verhasselt B., van Vlierberghe H., De Vos M., Raes J. (2021). Fecal Microbiota Transplantation Reduces Symptoms in Some Patients with Irritable Bowel Syndrome with Predominant Abdominal Bloating: Short- and Long-term Results from a Placebo-Controlled Randomized Trial. Gastroenterology.

[119] de Groot P., Nikolic T., Pellegrini S., Sordi V., Imangaliyev S., Rampanelli E., Hanssen N., Attaye I., Bakker G., Duinkerken G. (2021). Faecal microbiota transplantation halts progression of human new-onset type 1 diabetes in a randomised controlled trial. Gut.

[120] Karjalainen E.K., Renkonen-Sinisalo L., Satokari R., Mustonen H., Ristimaki A., Arkkila P., Lepisto A.H. (2021). Fecal Microbiota Transplantation in Chronic Pouchitis: A Randomized, Parallel, Double-Blinded Clinical Trial. Inflamm. Bowel Dis..

[121] Ianiro G., Bibbo S., Porcari S., Settanni C.R., Giambo F., Curta A.R., Quaranta G., Scaldaferri F., Masucci L., Sanguinetti M. (2021). Fecal microbiota transplantation for recurrent C. difficile infection in patients with inflammatory bowel disease: Experience of a large-volume European FMT center. Gut Microbes.

[122] Hanssen N.M.J., Nieuwdorp M. (2021). Fecal microbiota transplantation and fiber supplementation, better together?. Cell Rep. Med..

[123] El-Salhy M., Valeur J., Hausken T., Gunnar Hatlebakk J. (2021). Changes in fecal short-chain fatty acids following fecal microbiota transplantation in patients with irritable bowel syndrome. Neurogastroenterol. Motil..

[124] Segal A., Zlotnik Y., Moyal-Atias K., Abuhasira R., Ifergane G. (2021). Fecal microbiota transplant as a potential treatment for Parkinson’s disease—A case series. Clin. Neurol. Neurosurg..

[125] Wu L.H., Ye Z.N., Peng P., Xie W.R., Xu J.T., Zhang X.Y., Xia H.H., He X.X. (2021). Efficacy and Safety of Washed Microbiota Transplantation to Treat Patients with Mild-to-Severe COVID-19 and Suspected of Having Gut Microbiota Dysbiosis: Study Protocol for a Randomized Controlled Trial. Curr. Med. Sci..

[126] Ding D., Yong H., You N., Lu W., Yang X., Ye X., Wang Y., Cai T., Zheng X., Chen H. (2022). Prospective Study Reveals Host Microbial Determinants of Clinical Response to Fecal Microbiota Transplant Therapy in Type 2 Diabetes Patients. Front. Cell Infect. Microbiol..

[127] Huang C., Huang Z., Ding L., Fu Y., Fan J., Mei Q., Lou L., Wang J., Yin N., Lu Y. (2022). Fecal microbiota transplantation versus glucocorticoids for the induction of remission in mild to moderate ulcerative colitis. J. Transl. Med..

[128] Su L., Hong Z., Zhou T., Jian Y., Xu M., Zhang X., Zhu X., Wang J. (2022). Health improvements of type 2 diabetic patients through diet and diet plus fecal microbiota transplantation. Sci. Rep..

[129] Haifer C., Paramsothy S., Kaakoush N.O., Saikal A., Ghaly S., Yang T., Luu L.D.W., Borody T.J., Leong R.W. (2022). Lyophilised oral faecal microbiota transplantation for ulcerative colitis (LOTUS): A randomised, double-blind, placebo-controlled trial. Lancet Gastroenterol. Hepatol..

[130] Mazzawi T., Hausken T., El-Salhy M. (2022). Changes in colonic enteroendocrine cells of patients with irritable bowel syndrome following fecal microbiota transplantation. Scand. J. Gastroenterol..

[131] Ma Y., Liu J., Rhodes C., Nie Y., Zhang F. (2017). Ethical Issues in Fecal Microbiota Transplantation in Practice. Am. J. Bioeth..

[132] Baunwall S.M.D., Terveer E.M., Dahlerup J.F., Erikstrup C., Arkkila P., Vehreschild M.J., Ianiro G., Gasbarrini A., Sokol H., Kump P.K. (2021). The use of Faecal Microbiota Transplantation (FMT) in Europe: A Europe-wide survey. Lancet Reg. Health Eur..

[133] Maida M., McIlroy J., Ianiro G., Cammarota G. (2018). Faecal Microbiota Transplantation as Emerging Treatment in European Countries. Adv. Exp. Med. Biol..

[134] Sachs R.E., Edelstein C.A. (2015). Ensuring the safe and effective FDA regulation of fecal microbiota transplantation. J. Law Biosci..

